# Effect of silhouetting and inversion on view invariance in the monkey inferotemporal cortex

**DOI:** 10.1152/jn.00008.2017

**Published:** 2017-04-05

**Authors:** N. Apurva Ratan Murty, S. P. Arun

**Affiliations:** Centre for Neuroscience, Indian Institute of Science, Bangalore, India

**Keywords:** object recognition, shape coding, invariance, viewpoint

## Abstract

We effortlessly recognize objects across changes in viewpoint, but we know relatively little about the features that underlie viewpoint invariance in the brain. Here, we set out to characterize how viewpoint invariance in monkey inferior temporal (IT) neurons is influenced by two image manipulations—silhouetting and inversion. Reducing an object into its silhouette removes internal detail, so this would reveal how much viewpoint invariance depends on the external contours. Inverting an object retains but rearranges features, so this would reveal how much viewpoint invariance depends on the arrangement and orientation of features. Our main findings are *1*) view invariance is weakened by silhouetting but not by inversion; *2*) view invariance was stronger in neurons that generalized across silhouetting and inversion; *3*) neuronal responses to natural objects matched early with that of silhouettes and only later to that of inverted objects, indicative of coarse-to-fine processing; and *4*) the impact of silhouetting and inversion depended on object structure. Taken together, our results elucidate the underlying features and dynamics of view-invariant object representations in the brain.

**NEW & NOTEWORTHY** We easily recognize objects across changes in viewpoint, but the underlying features are unknown. Here, we show that view invariance in the monkey inferotemporal cortex is driven mainly by external object contours and is not specialized for object orientation. We also find that the responses to natural objects match with that of their silhouettes early in the response, and with inverted versions later in the response—indicative of a coarse-to-fine processing sequence in the brain.

## Abstract (precis)

We easily recognize objects across changes in viewpoint, but the underlying features are unknown. Here, we show that view invariance in monkey inferotemporal cortex is driven mainly by external object contours and is not specialized for object orientation. We also find that the responses to natural objects match with that of their silhouettes early in the response, and with inverted versions later in the response—indicative of a coarse-to-fine processing sequence in the brain.

we easily recognize objects across rotations in depth, but the features that enable such viewpoint-invariant recognition remain poorly understood. An obvious approach would be to study how viewpoint invariance is affected by systematic image manipulations. This approach has, for instance, yielded many insights into animal categorization (Delorme et al. 2000; Girard and Koenig-Robert 2011; Joubert et al. 2009; Harel and Bentin 2009; Mohan and Arun 2012; Morrison and Schyns 2001; Wichmann et al. 2006, 2010). Yet it has not been used to study view-invariant object representations in the brain. Our goal was to elucidate the features underlying view invariance in the brain through systematic image manipulations. We targeted the monkey inferior temporal (IT) cortex, an area critical for object recognition whose neurons are invariant to size, position, and viewpoint (Connor et al. 2007; DiCarlo et al. 2012; Tanaka 1992). We selected two image manipulations: silhouetting and inversion.

Silhouetting is interesting because it removes all internal detail. Therefore, it would show how much view invariance is based on external contours alone. In the extreme, view invariance might be abolished if it were based only on internal detail or texture, but there is reason to believe that view invariance might be present even for silhouettes, since many objects can be recognized despite silhouetting (Mitsumatsu and Yokosawa 2002; Panis et al. 2008; Quinn et al. 2001). There have been many examples of individual neurons showing similar responses to objects and silhouettes (Kayaert et al. 2003; Ratan Murty and Arun 2015; Tsunoda et al. 2001; Yamane et al. 2006, 2008), and this is true on average as well (Kovács et al. 2003). Although we have shown recently that view invariance is weaker for silhouettes (Ratan Murty and Arun 2015), it is not clear whether the same neurons show view invariance across natural and silhouette images.

Inverting an image is interesting because it preserves image features but alters their orientation and arrangement. If view invariance were specialized for features in upright objects such as animals, it would be diminished for inverted objects. This is consistent with the observation that object classification is slower on inverted objects (Guyonneau et al. 2006; Sekuler et al. 2004). The impact of inversion has been studied in great detail for faces, whose recognition is strongly affected by inversion (Tsao and Livingstone 2008). Face-selective neurons in IT show strong response modulation with inversion (Sugase-Miyamoto et al. 2014; Taubert et al. 2015). Similar findings have been reported in the face patch network (Freiwald et al. 2009; Freiwald and Tsao 2010; Taubert et al. 2015). However, while individual IT neurons show strong tuning for orientation (Ashbridge et al. 2000; Rollenhagen and Olson 2000; Yamane et al. 2006), the impact of inversion in general on neuronal selectivity and view invariance in IT has not been systematically investigated.

Here, we investigated how view invariance and selectivity of IT neurons is affected by silhouetting and inversion. We tested each object in two disparate views to test many objects across these image manipulations. However, our results are likely to hold across smaller viewpoint changes since the image itself does not change much, and across larger viewpoint changes, that may result in similar responses in IT neurons due to mirror confusion (Rollenhagen and Olson 2000). Our main findings are that view invariance is reduced by silhouetting but not inversion and that IT neurons generalize early across silhouetting and only later to inversion. These findings place constraints on models of view invariance.

## METHODS

All experiments were performed in accordance to a protocol approved by the Institutional Animal Ethics Committee of the Indian Institute of Science, Bangalore, and the Committee for the Purpose of Control and Supervision of Experiments of Animals, Government of India. All experimental procedures are identical to those described in previous studies from our laboratory (Ratan Murty and Arun 2015, 2017). Therefore, only details specific to this study are described below.

### Neurophysiology

We recorded extracellular activity from 126 visual neurons in the anterior inferotemporal cortex of four monkeys (54 from monkey *Ka*, 31 from monkey *Sa*, 15 from monkey *Ro*, and 26 from monkey *Xa*). In all animals, the recording sites were determined to be in the anterior ventral inferotemporal cortex. Structural MRI images of the recording sites are available in previous studies performed on the same animals: monkeys *Ro* and *Xa* (Ratan Murty and Arun 2015) and monkeys *Ka* and *Sa* (Ratan Murty and Arun 2017). Recordings were obtained using either single electrodes in monkeys *Ro* and *Xa* (Ratan Murty and Arun 2015) and using 24-channel multicontact electrodes in monkeys *Ka* and *Sa* (Ratan Murty and Arun 2017). The key results reported in this study were similar for data from each animal considered separately. A subset of this data has been published in a previous study (Ratan Murty and Arun 2015).

### Behavioral Task

All animals performed a fixation task. Each trial began with the appearance of a fixation dot on which the animal had to fixate. On successful fixation, the animal had to maintain its gaze within a 3° window of the dot, while a series of eight images were flashed on the screen for 200 ms each, with an equal interstimulus duration, following which it received a juice reward. Even though the fixation window was relatively large, our post hoc analyses showed that, across animals, recording sessions, and trials, the eye positon was very closely centered at fixation (standard deviation of eye position across the trial: 0.23° for horizontal and 0.34° for vertical on average across all trials and sessions).

### Stimuli

**Fig. 1.**
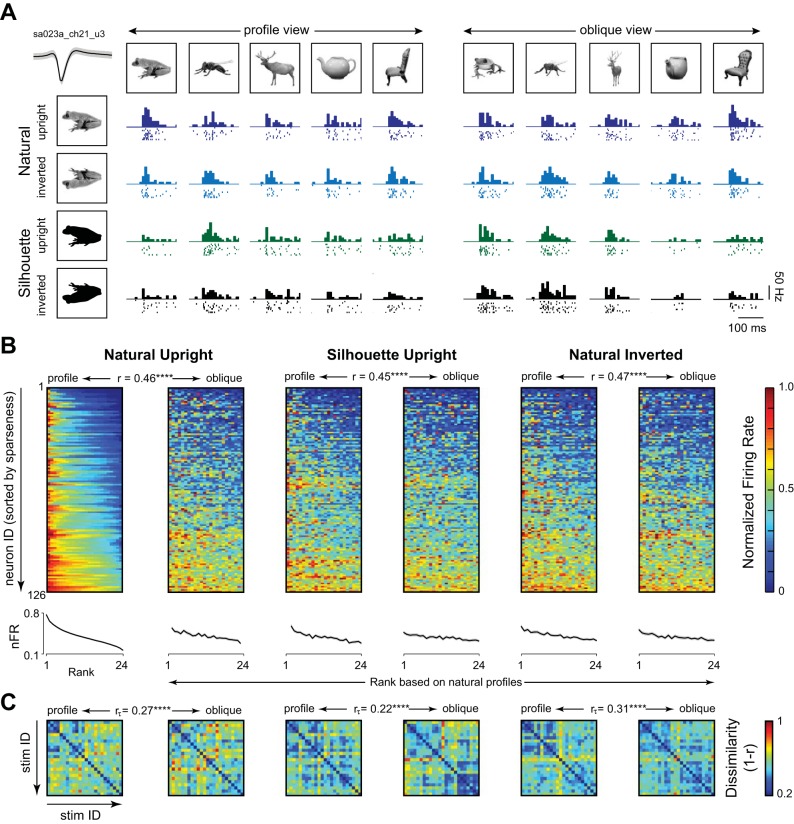
Effect of silhouetting and inversion on inferior temporal (IT) neurons. *A*: responses of an example IT neuron to a subset of four objects in two views, across silhouetting and inversion in the image-on period (200 ms). Ticks indicate single action potentials, and the histogram bars represent the firing rate of the neuron in 10-ms bins. Silhouettes are depicted here as black against white, but in the actual experiment, they were white against a black background. The top left inset depicts the mean waveform shape (thick line) and standard deviation across all detected waveforms (gray shading). *B*, *top:* normalized response for each neuron plotted for objects ranked according to its preference in the natural upright profile images. Neurons are sorted by response sparseness calculated across the natural profile stimuli. The correlation coefficients above the color maps represent the match between the profile and oblique response maps and are, therefore, indicative of view invariance. ****P < 0.00005. *Bottom:* average normalized response (nFR, black) across neurons to stimuli ranked in the same manner, with shaded regions representing the SE across neurons. *C*: pairwise neural dissimilarity (measured as correlation distance, 1 − *r*) between all stimuli in each condition. The order of stimuli is the same as in Fig. 6. The correlation coefficients above these dissimilarity matrices represent the match between the profile and oblique population representations and are, therefore, also indicative of view invariance. The match was calculated using the Kendall’s Tau rank-order correlation coefficient, a robust measure of correlation when samples are not independent (as is the case here, since the same neural responses are used to calculate all pairs of dissimilarities).

We selected 24 naturalistic objects from a larger set tested in our previous study (Ratan Murty and Arun 2015). These comprised 12 animate and 12 inanimate objects each at two viewpoints (a profile view and a ~45° depth-rotated view). We selected two viewpoints per objects to study the four possible image manipulations (natural/silhouette × upright/inverted) across many objects. For each image, we created a silhouette, inverted, and inverted-silhouette versions (Fig. 1). The silhouette version was created by thresholding the original image and using the Live Trace function (Adobe Illustrator, Adobe). The inverted version was created by reflecting the original about the horizontal axis. The inverted-silhouette version was simply the inversion version of the silhouette. Stimuli were scaled, such that the longer dimension of the profile view was 5.5°, and the corresponding oblique view was equated to have the same height, such that it appeared to be a plausible rotation in depth of the profile view. Thus, in all, there were 48 images (24 objects × 2 views) in four possible variations: natural-upright, natural-inverted, silhouette-upright, and silhouette-inverted, resulting in a total of 192 stimuli tested on each neuron.

### Trial Design

The stimuli were presented in pseudo-random order with the constraint that two views of a given object never occurred together in a single trial. Each stimulus was presented at least eight times over the course of a recording session. To avoid drastic changes in stimulus intensity within a single trial, all four groups of stimuli i.e., upright, inverted, silhouette-upright, and silhouette-inverted stimuli were presented in separate blocks in randomized order across sessions.

### Pixel and Visual Cortex Representations

To investigate how silhouetting and inversion might influence the inputs to IT, we tested two low-level visual representations: a pixel model that mimics the retinal input, and a visual cortex (V1) model that mimics the input to IT from the primary visual cortex. In the pixel model, the representation of each 200 × 200 pixel image is simply its pixel intensities concatenated into a single vector with 40,000 dimensions. In the V1 model, the representation of each image is the concatenated output of a bank of Gabor filters tuned to spatial frequency and orientation at each location, with input- and output-divisive normalization, as we have described previously (Ratan Murty and Arun 2015). The V1 model had 1,920,000 outputs, consisting of filters spanning six spatial frequencies, each at eight orientations at each pixel location.

### Sparseness

We used a standard measure of response sparseness to quantify the selectivity for each neuron (Zhivago and Arun 2016; Zoccolan et al. 2007). For a neuron with responses *r_1_ r_2_ r_3_… r_n_* to *n* stimuli in a set, its sparseness is defined as

$$S=\left[1-{\left(\sum r_{i}/n\right)}^{2}/\left(\sum r_{i}{}^{2}/n\right)\right]/\left(1-1/n\right)$$

This measure ranges from 0 for equal firing to all stimuli to 1 for exclusive firing to only one stimulus in a set. We reported the sparseness for each neuron across the responses to natural profile views in the results, but obtained similar results using oblique views as well.

### Population Decoding

We used a population-decoding analysis to measure how well the neuronal population signals object-identity across image transformations (silhouetting, inversion, or viewpoints). For each trial (*n* = 8 trials), stimulus (*n* = 192 stimuli), and time bin (20 ms), we created a population-response vector containing the firing rate of each neuron. This step assumes that neurons were recorded simultaneously, but since their trial-wise firing rates were independently recorded at different times, the resulting decoding accuracy becomes an upper bound on the performance expected with simultaneous recordings (Hung et al. 2005). We then projected the response vectors along their principal components to retain 80% of the variance. This step was necessary, especially in the early parts of the response when many neurons had almost no response, resulting in infinitely many solutions for the best hyperplane-separating responses from different groups. Varying this fraction did not change the results qualitatively.

#### View-invariant object decoding.

**Fig. 2.**
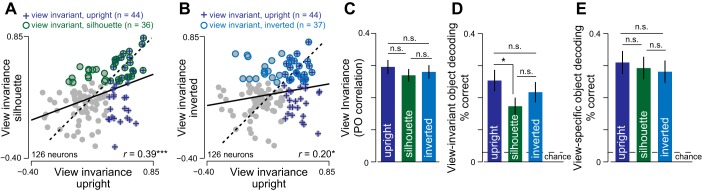
Effect of silhouetting and inversion on viewpoint invariance. *A*: scatterplot of view invariance (profile-oblique correlation across 24 objects) for silhouette vs. natural-upright objects across neurons. The dotted line is the *y* = *x* line, and the solid line is the best-fitting line. Green circles indicate cells with significant view invariance for silhouettes (profile-oblique correlation, *P* < 0.05), and blue crosses indicate cells with significant view invariance for upright objects. *B*: scatterplot of view invariance for inverted vs. upright objects across neurons. Conventions are as in *A*. *C*: view invariance for upright, silhouette, and inverted objects. Error bars indicate the SE across neurons. *D*: view-invariant object decoding accuracy for upright, silhouette, and inverted objects, calculated across 192 trials (24 objects × 8 trials). The dashed line indicates chance performance for the object decoder (1/24 = 4.2%). *P < 0.05. ***P < 0.0005. n.s., not significant. Error bars indicate the means ± SE of the decoding accuracy across trials. *E*: view-specific object decoding performance for upright, silhouette, and inverted objects. The dashed line indicates the chance performance for the object decoder (1/24 = 4.2%). Error bars indicate the means ± SE of the decoding accuracy across trials.

We measured view invariance by asking how well the recorded population of neurons could signal object identity across viewpoint. To this end, we trained a linear classifier (*classify* function, MATLAB v2014a) on single-trial responses and the associated object labels in the profile view and tested it on the responses to the oblique view (and vice versa; see Fig. 2*D*). Because this classifier has to identify the correct object out of 24 possible objects, chance performance is 1/24 = 4.1%.

#### View-specific object decoding.

To establish a baseline for how well the population of neurons could signal object identity at a particular view, we trained a linear classifier on the single-trial responses to one view alone on all but one trial and tested its accuracy on the left-out trial (see Fig. 2*E*). Because this classifier has to identify the correct object out of 24 possible objects, chance performance for this classifier is 4.1%.

#### Silhouette/inversion invariant object decoding.

To measure the ability of IT neurons to signal object identity across silhouetting or inversion, we trained a linear classifier on single-trial responses for natural upright objects and tested it on the responses to silhouetted objects or inverted objects (Fig. 4*C*). Because this classifier has to identify the correct object out of 24 possible objects, chance performance for this classifier is 4.1%.

## RESULTS

We recorded from 126 visually responsive neurons from the left IT cortex of four monkeys performing a fixation task. Each neuron was tested using 24 naturalistic objects (12 animate, 12 inanimate) shown in two viewpoints (profile and oblique) across four image manipulations (natural/silhouette × upright/inverted). We tested only two viewpoints to test many objects across all four image manipulations. The objects used were a subset of those used in an earlier study (Ratan Murty and Arun 2015). In our earlier study, we have characterized the baseline invariance expected from image pixels, the view invariance observed in IT neurons, the dynamic transition from early-view sensitivity to late-view invariance, and compared view invariance in IT with several computational models. For these details, we refer the reader to our previous study (Ratan Murty and Arun 2015). Here, we focus on the effect of silhouetting and inversion on viewpoint invariance in IT neurons.

The responses of an example IT neuron to silhouetting and inversion of a subset of objects are shown in Fig. 1*A*. It can be seen that the neuron maintains its preference for objects across views for all four groups: upright, inverted, silhouette-upright, and silhouette-inverted images. To quantify its view invariance, we calculated the correlation between the mean firing rate (in a 50–200-ms window) across objects in the profile and oblique views. This revealed a positive correlation for this neuron (*r* = 0.47, 0.43, 0.26, and 0.26 for upright, inverted, silhouette-upright, and silhouette-inverted images, respectively; *P* < 0.05 for upright and inverted natural objects, and *P* = 0.21 and 0.22 for upright and inverted silhouettes). Across neurons, roughly a one-third of all cells showed significant response correlation across views (average significant correlation: 0.44 across 44 neurons for natural upright; 0.58 across 37 neurons for natural inverted; 0.54 across 36 neurons for silhouette-upright; 0.54 across 31 neurons for silhouette-inverted). This fraction of view-invariant cells is lower than the fraction reported in our earlier study, but the difference could arise because of fewer objects tested in this study, reducing the ability to detect a significant correlation.

To visualize view invariance for all neurons, we first normalized the responses of each cell by its maximum response across all stimuli across conditions. We then ranked the responses of each neuron to natural objects in their profile view from best to worst, and we sorted stimuli in the same order for the oblique view, and for both views in the silhouette and inverted versions. In the resulting color maps (Fig. 1*B*), it can be seen that neurons continue to respond to their preferred object across viewpoint and across silhouetting and inversion as well. This is even more evident upon taking the population average firing rates, which declines monotonically for both views in all conditions, even though the object preference was determined only using the natural profile views (Fig. 1*B*, *bottom*). These response matrices also showed a significant correlation across views, indicative of widespread view invariance (*r* = 0.46, 0.45, and 0.47 for upright, silhouette, and inverted, *P* < 0.00005; Fig. 1*B*). To visualize the view invariance for the entire population as a whole, we calculated the neural dissimilarity for all pairs of objects at a particular view (calculated for each object pair as one minus the correlation between activity evoked by the two objects across all neurons). The neural dissimilarities at one view were significantly correlated with those at the other view, again indicative of viewpoint invariance (Fig. 1*C*; Kendall’s Tau rank-correlations: 0.27, 0.22, and 0.31 for natural, silhouette and inverted versions; *P* < 0.00005, permutation test).

### Does View Invariance Change with Silhouetting or Inversion?

We then compared view invariance for natural upright objects with silhouette and inverted versions. To investigate whether view invariance is affected by silhouetting, we plotted the view invariance for silhouette objects against that of natural objects. Interestingly, this revealed a positive correlation (*r* = 0.39, *P* < 0.00005; Fig. 2*A*). This correlation remained significant, even after regressing out the overall sparseness of the neural responses across stimuli (*r* = 0.37, *P* < 0.00005). Thus, neurons that are view invariant for natural objects show view invariance for their silhouettes too. The observed overlap between neurons that show view invariance for both upright and silhouette conditions is greater than expected, given their individual rates of incidence (number of neurons with significant view invariance for both conditions: *n* = 23; expected overlap given 44/126 view-invariant neurons in upright and 36/126 view-invariant neurons in silhouette conditions: *n* = 12; *P* < 0.05, χ^2^ test). Because natural images and silhouettes only share external contours, this result implies that view invariance is probably driven by external contours rather than internal details, at least for the objects tested. On comparing the magnitudes of invariance, however, we found that view invariance was slightly weaker for silhouettes compared with natural objects, but this difference did not reach statistical significance (average view invariance across all neurons: 0.30 and 0.24 for upright and silhouette, *P* = 0.07, Wilcoxon signed-rank test; Fig. 2*C*). This lack of effect persisted even across neurons with significant profile-oblique correlations (*P* < 0.05) in each condition (average significant correlation: 0.57 across 44 neurons for upright; 0.54 across 36 neurons for silhouette, *P* = 0.57, Wilcoxon rank-sum test).

We next analyzed the impact of inversion on view invariance by plotting the view invariance for inverted objects against that of natural objects. This too revealed a positive correlation (*r* = 0.20, *P* < 0.05; Fig. 2*B*). This correlation remained significant, even after regressing out the overall sparseness of the neural response across stimuli (*r* = 0.19, *P* < 0.05). In other words, neurons that are view invariant for natural objects are also view invariant for inverted objects. The observed overlap between view-invariant neurons in both conditions (*n* = 21) was significantly greater than the overlap expected, given their independent rate of incidence (expected overlap: *n* = 12; *P* < 0.05, χ^2^ test). This result implies that view invariance may depend on inversion-independent features at least for the objects tested. As with silhouettes, we did not see a significant difference in view invariance across inversion (average view invariance: *r* = 0.30 and 0.29 for upright and inversion, *P* = 0.75, Wilcoxon signed-rank test; Fig. 2*C*). This lack of effect persisted even across neurons with significant profile-oblique correlation (*P* < 0.05) in each condition (average significant correlation: 0.57 across 44 neurons for upright; 0.58 across 37 neurons for inverted, *P* = 0.72, Wilcoxon rank-sum test).

### Does View-Invariant Decoding Change with Silhouetting or Inversion?

The above analyses characterized the average invariance across all cells, but a more efficient decoding mechanism might depend more on cells that are more invariant. To this end, we performed a population-decoding analysis to estimate how well the entire neural population can represent object identity in a view-invariant manner. To this end, we trained a linear classifier using the single-trial responses of all neurons (see methods) to objects at one view (e.g., profile) and tested it on responses to the other view (e.g., oblique). The accuracy of this classifier, which we denote as view-invariant decoding, is a measure of how well the neural population signals object identify independent of viewpoint. The results of this analysis are shown in Fig. 2*D*. View-invariant decoding accuracy for all image manipulations was significantly above chance (average accuracy: 25%, 18%, and 21% for upright, silhouette, and inverted conditions, respectively; *P* < 0.00005, one-tailed binomial test on correct/incorrect labels across 192 trials in each condition). View-invariant decoding was significantly weaker for silhouettes compared with upright objects (*P* < 0.05, Wilcoxon rank-sum test on trial-wise decoding accuracy; Fig. 2*D*). However, view invariance for inverted objects was not significantly different compared with upright objects (*P* = 0.25, Wilcoxon rank-sum test on trial-wise decoding accuracy; Fig. 2*D*). We conclude that silhouetting, but not inversion, reduces view invariance in IT neurons.

### Could Differences in View Invariance Arise from Differences in Selectivity?

The above differences in view invariances could arise simply if neurons were less selective for objects in one image manipulation over another. We assessed this possibility in several ways. First, we calculated the ability of IT neurons as a population to signal object identity at a particular view using a view-specific object decoder (see methods). This gives us an estimate of how well objects can be classified at a single view. We observed higher classification performance of objects when trained and tested at the same view (as expected), but importantly, no significant differences in view-specific object decoding across upright/inverted/silhouette conditions (Fig. 2*E*).

Second, we asked whether IT neurons show differences in selectivity across upright, silhouette, and inverted conditions. To this end, we calculated for each neuron its sparseness across the profile views in each condition and asked whether sparseness differed across conditions. This revealed no significant difference (average sparseness: 0.29, 0.30, and 0.26 for upright, silhouette, and inverted profile views; *P* > 0.1, Wilcoxon rank-sum test for all pairs of comparisons). Third, we asked whether IT neurons show differences in their reliability of firing across the three conditions. To this end, we calculated for each neuron the correlation between the average firing rates calculated using two halves of the recorded trials. Here, too, we found no significant differences between the three image manipulations (split-half correlations: 0.36 ± 0.02, 0.36 ± 0.02 and 0.37 ± 0.02, all pairs of comparisons *P* > 0.1, Wilcoxon rank-sum test across neurons).

Thus, population decoding, selectivity, or reliability do not change with silhouetting or inversion, and, therefore, cannot explain the observed difference in view invariance.

### Is View Invariance Specialized for Animate Objects?

Next, we asked whether view invariance was different between the animate and inanimate objects tested in this study. We speculated that if object representations are specialized for animate objects in general despite no explicit familiarization on our part, we should find greater view invariance for animate compared with inanimate objects. We observed no such difference in the magnitude of view invariance (average profile-oblique correlation: *r* = 0.25 for animates and 0.25 for inanimates; *P* = 0.75, Wilcoxon signed-rank test), or in view-invariant decoding (average accuracy: 29% for animates and 25% for inanimates; *P* = 0.29, Wilcoxon rank-sum test on trial-wise decoding accuracy). We also compared view invariance for animate objects across inversion, and here, too, we observed no systematic difference (average profile-oblique correlation: *r* = 0.25 for upright, *r* = 0.23 for inverted, *P* = 0.65, Wilcoxon signed-rank test across neurons; view-invariant decoding accuracy: 25% for animate upright, 22% for animate inverted, *P* = 0.24, Wilcoxon rank-sum test on trial-wise decoding accuracy). We conclude that, at least for the objects tested and with no explicit familiarization, there is no special or stronger view invariance for animate objects.

### Do View-Invariant Neurons Also Generalize Across Silhouetting and Inversion?

**Fig. 3.**
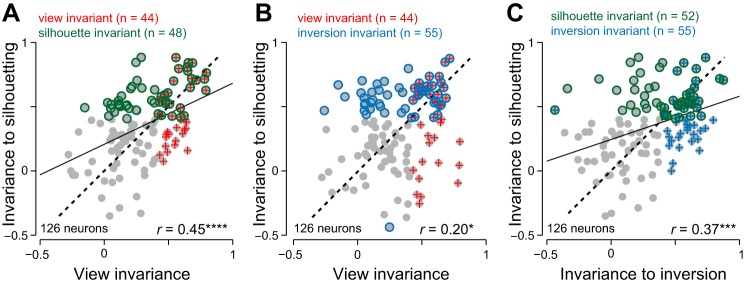
Invariance to silhouetting and inversion. *A*: silhouetting invariance for each neuron (measured as the response correlation between natural upright images and silhouette upright images in the profile view across 24 objects) plotted against view invariance (response correlation between natural upright profile and oblique views across 24 objects) for each neuron. Conventions are as in Fig. 2*A*. *B*: inversion invariance (measured as the response correlation between natural upright and inverted images in the profile view) plotted against view invariance. *C*: silhouetting invariance plotted against inversion invariance. *P < 0.05. ***P < 0.0005. ****P < 0.00005.

Next, we asked whether neurons that show view invariance also generalize across silhouetting and inversion. To assess this possibility, we calculated for each neuron the degree of invariance to silhouetting as the correlation in its response to objects in their profile view with their silhouette versions. We avoided including both views to avoid any artefactual correlation arising from using the same data twice, but we obtained similar results on including both views or using only the oblique view (data not shown). We then plotted the invariance to silhouetting against view invariance as before (*r =* 0.45, *P* < 0.00005, Fig. 3*A*). In a similar fashion, we plotted the invariance to inversion against view invariance—this too revealed a positive correlation (*r* = 0.20, *P* < 0.05, Fig. 3*B*). Both correlations remained significant, even after factoring out the overall sparseness of the neural response (*r* = 0.42, *P* < 0.00005, and *r* = 0.19, *P* < 0.05, respectively, for silhouette-view invariance and inversion view-invariance). We further asked whether there was any relation between cells that show invariance to silhouetting and those that show invariance to inversion. This too revealed a significant correlation (*r* = 0.37, *P* < 0.0005; Fig. 3*C*).

These findings can be interpreted in two ways: first, strong view invariance may depend on external contours and inversion-independent features. Alternatively, neurons that are invariant may be invariant across a number of properties, including viewpoint, silhouetting, and inversion. This is consistent with the idea that invariance may be an intrinsic property for a neuron that covaries with its selectivity (Zhivago and Arun 2016).

### Dynamics of Generalization to Silhouetting and Inversion

**Fig. 4.**
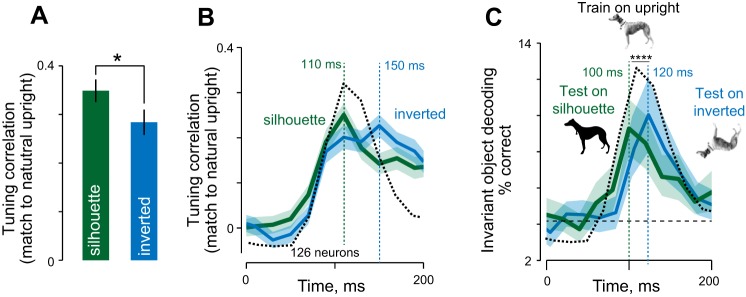
Effect of silhouetting and inversion on object representations in IT. *A*: tuning correlation between natural upright and their silhouettes in the profile view across 24 objects (green) and between natural upright and inverted in the profile view (blue). *P < 0.05. *B*: time course of tuning correlation in 20-ms bins between natural and silhouette objects in the profile view (green) and between natural and inverted objects in the profile view (blue). The bold trace indicates the mean, and the shaded regions indicate the means ± SE across neurons. The black dashed line indicates the mean normalized response across all the stimuli overlaid for comparison. *C*: time course of decoding accuracy of a classifier trained on natural upright objects (profile views) and tested on their silhouettes (green) and inverted versions (blue). The bold trace indicates the mean, and shaded regions represent standard deviation over 100 bootstrap-derived time course estimates obtained by sampling 100 cells with replacement. The dashed line indicates chance performance of object decoding (1/24 = 4.2%), and the triangles below indicate the peak latency. The black dotted curve indicates the mean normalized response across all the stimuli overlaid for comparison. ****P < 0.00005.

Next, we asked whether neurons generalize better across silhouetting or inversion. To assess this possibility, we compared the magnitude of invariance to silhouetting with the invariance to inversion. Across neurons, we observed a stronger invariance for silhouettes compared with inversion (average invariance: *r* = 0.34 and 0.23 for silhouetting and inversion respectively, *P* < 0.05, Wilcoxon signed-rank test; Fig. 4*A*).

To examine the dynamics of generalization to silhouette and inverted versions, we repeated this analysis on 20-ms bins over the course of the visual response. We found that the invariance across silhouetting peaks early (*t* = 110 ms) followed by invariance to inversion (*t* = 150 ms), although this difference in latency did not reach statistical significance (*P* = 0.30, Wilcoxon signed-rank test on peak latency across neurons; Fig. 4*B*). Although the magnitude of invariance across inversion and silhouetting did not differ significantly at *t* = 110 ms (*P* = 0.19, Wilcoxon signed-rank test comparing the tuning correlations between upright and silhouette, and upright and silhouette across neurons), invariance across inversion was stronger than invariance across silhouetting at *t* = 150 ms (*P* < 0.05, Wilcoxon signed-rank test comparing the tuning correlations between upright and silhouette, and upright and silhouette across neurons).

The above analyses indicate the average degree of generalization across neurons but a more efficient decoder might prioritize the more invariant neurons, giving rise potentially to different dynamics. To this end, we performed a population decoding analysis exactly as before, but this time we trained an object classifier on the profile views of all natural upright objects, and then tested it on silhouettes and inverted objects. This revealed an interesting dynamic transition in the neuronal representation: across the population, object identity could be decoded for silhouettes early in the response (peak latency = 100 ms), and only 20 ms later on for inverted objects (peak latency = 120 ms; Fig. 4*C*). To assess the statistical significance of this latency difference, we performed a bootstrap analysis. We sampled 100 neurons with replacement each time and calculated the peak-decoding latency for silhouettes and inverted objects each time. We then determined the significance to be the fraction of times the silhouette-decoding latency was larger than the inverted-decoding latency. This revealed the latency difference to be significant (*P* = 0.04). We conclude that the object representation in IT matches early with silhouettes and only later on with inverted objects.

The above differences in dynamics of silhouetting and inversion could have arisen simply from latency differences in the neural responses for these groups. To assess this possibility, we calculated the time at which the response of each neuron attained its peak for natural (upright) objects, silhouettes, and inverted objects. The response latency for these groups did not differ significantly (average peak latency across neurons: 118, 115, and 119 ms for upright, silhouette and inverted; *P* > 0.05 for all pairwise comparisons, Wilcoxon signed-rank test across neurons). Thus, the early match between natural and silhouette objects and the later match with inverted cannot be explained using differences in response latency.

We next considered whether there are neurons selective only for silhouettes or natural images. Although this is unlikely given that the population generalizes across silhouetting, we nonetheless assessed this possibility by calculating a modulation index of the form (*S* − *N*)/(*S* + *N*), where *S* and *N* are the average responses to silhouettes and naturals, respectively across both object views. The median absolute value of this modulation index was 0.08 ± 0.01, indicating only moderate selectivity for one category over another. There were only two neurons with a modulation index larger than one-third, indicative of twice as strong a response for silhouettes over naturals and conversely only three neurons with a modulation index less than −1/3, indicative of a greater response to naturals over silhouettes. Thus, very few neurons show exclusive responses to natural and silhouette images but typically respond to both image categories.

### Could the Observed Results Be Explained Using Low-Level Image Representations?

**Fig. 5.**
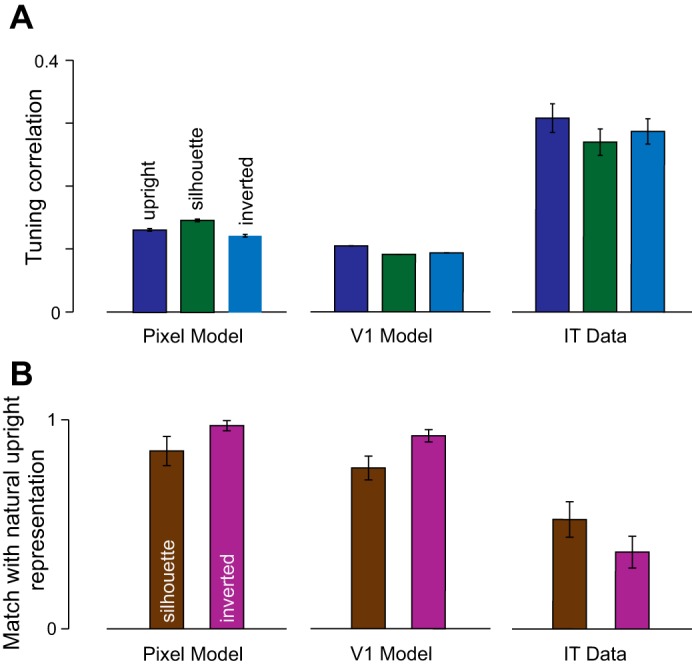
Baseline invariance and effect of silhouetting and inversion on low-level visual representations. *A*: average tuning correlation between profile and oblique views across pixel, V1, and IT units. Error bars indicate the means ± SE across units. Note that the IT data here are the same as in Fig. 2*C*. *B*: to establish a baseline for the effect of silhouetting and inversion on IT neurons, we calculated pairwise distances between natural upright objects in their profile views and compared this with the pairwise distances between their silhouette or inverted versions using the correlation coefficient. This measure has the advantage that it is scale-free and can be used to compare any two representations. This measure is plotted for the pixel model, V1 model, and data from IT neurons. In each case, error bars represent bootstrap-derived estimates of standard deviation obtained by resampling 80% of all pixels, model units, or neurons (for pixel, V1, and IT) with replacement.

Next, we asked whether the results observed in IT neurons could be a trivial consequence of low-level image representations. To this end, we investigated two low-level representations: a pixel model and a V1 model (see methods). First, we asked whether the view invariance observed in the pixel and V1 models is comparable to that observed in IT. To this end, we measured the response correlation between the profile and oblique views just as we did with IT neurons. As expected, the tuning correlation observed for the V1 and the pixel models was much lower than observed in the IT (Fig. 5*A*).

Second, we asked whether the observed dynamic differences between silhouette and inversion can be explained by larger representational changes in one manipulation compared with the other. To establish a baseline for these dynamics, we calculated the effect expected from image pixels and from a V1 model and compared this with the effect observed in IT neurons. We compared the overall representation of the pixel and V1 models with IT neurons using dissimilarity relations between objects across all model units. To this end, we calculated for each model the pairwise distance between every pair of objects (in their profile view) for upright, silhouette, and inverted images. For pixels, we measured the distance between a pair of images using the sum of absolute differences in pixel-wise intensities across the image. For V1 model units, the distance between two images was likewise calculated as the average absolute differences between the activity of model units in response to the two images. For IT neurons, the distance between two images was calculated as the average absolute difference in firing rate evoked by the two images. We then compared the match between the upright and silhouette distances, and compared it with the match between upright and inverted distances (Fig. 5*B*). For pixels and V1, the inverted object representation matched better with the upright objects than the silhouette representation. This makes sense because the total change in the image is larger when the internal content of the image is removed than when it is inverted. In contrast, for IT neurons, silhouette distances were a better match to the natural upright object distances than were inverted object distances (Fig. 5*B*).

If natural upright and inverted images are more similar based on pixels or V1, this would predict a stronger and earlier match between upright and inverted than between natural and silhouettes. We observed the opposite pattern in IT neurons: responses to natural objects were similar to silhouettes early in the response and were similar to inverted objects only later in the response (Fig. 4). We interpret this as a coarse-to-fine processing scheme in IT, whereby neurons are initially sensitive to the external contour and only later to finer internal details.

### Does the Effect of Silhouetting and Inversion Depend on Object Structure?

We have previously shown that view invariance in IT neurons depends on the degree to which an object foreshortens across views (Ratan Murty and Arun 2015). Specifically, we found view invariance to be stronger for objects that did not foreshorten across views than for those that did. To reconfirm these findings in our data, we divided the objects into high and low foreshortening groups (as assessed by the change in width of the image between profile and oblique views; 12 objects each) and asked whether view-invariant decoding differed across the two groups. This revealed a similar pattern, as we have observed previously (Ratan Murty and Arun 2015): view-invariant decoding was stronger for the high foreshortening group (average view-invariant object decoding performance: 26% and 33% for the high and low foreshortening groups).

**Fig. 6.**
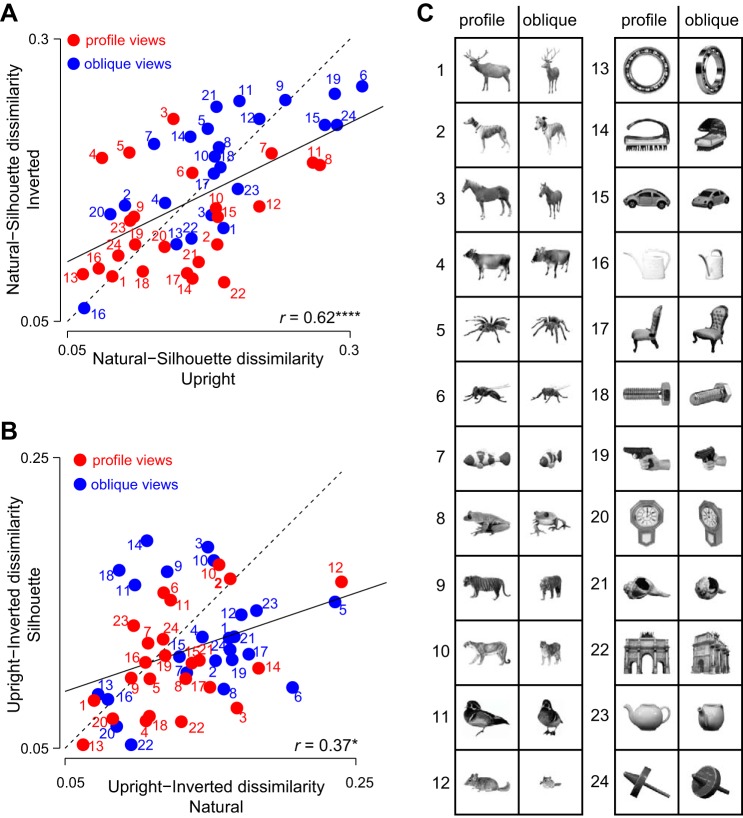
Object-wise effects of silhouetting and inversion. *A*: to calculate the impact of silhouetting for each object, we calculated the neural dissimilarity across neurons between the original and silhouette versions of that image. The neural dissimilarity is calculated as 1 − *r*, where *r* is the correlation coefficient between neural response vectors for the original image and its silhouette. We then plotted the natural-silhouette dissimilarity for inverted against upright stimuli for profile (red) and oblique views (blue) of each object. Numbers alongside each plot indicate stimulus identity shown in *C*. ****P < 0.00005. *B*: natural-inverted dissimilarity for silhouette against original stimuli for objects in their profile and oblique views, calculated as in *A*. *P < 0.05. *C*: stimuli used in the experiment, shown for natural upright objects, in both views. Numbers denote the objects shown in *A* and *B*.

Next, we investigated whether the effect of silhouetting and inversion on the neural response depends on object structure. For instance, oblique views of objects appear more degraded by silhouetting, whereas the profile views remain recognizable. To investigate this possibility, we calculated for each object the dissimilarity between the original image and the transformed image using the correlation distance across neurons (i.e., one minus the correlation between the responses evoked across the population to the original and transformed image). We then plotted the neural dissimilarity between original and silhouette images for inverted against upright objects (Fig. 6*A*). This revealed a positive correlation, indicating that the effect of silhouetting is similar for upright and inverted objects (*r* = 0.62, *P* < 0.00005). Additionally, as we had predicted, oblique views became more similar to each other due to silhouetting compared with profile views (Fig. 6*A*; average dissimilarity for upright objects: 0.14 for profile views, 0.19 for oblique views, *P* < 0.005; for inverted objects: 0.13 for profile and 0.18 for oblique, *P* < 0.005; Wilcoxon signed-rank test across 24 objects).

There are also systematic variations among objects, even in their profile views: for instance, neural dissimilarity between the deer (*object 1*) and its silhouette was small, whereas neural dissimilarity between the fly (*object 6*) and its silhouette was large. We speculate that this is because the three-dimensional shape of the deer is still evident in its silhouette, but this is not so for the fly. However, it is difficult to quantify these observations without more systematic analyses of how much three-dimensional shape information is retained in silhouettes. However, such patterns can be seen across all of the objects tested (Fig. 6*C*).

To investigate the effect of inversion on objects, we plotted the neural dissimilarity between the original and inverted images for silhouettes against natural objects (Fig. 6*B*). This too revealed a positive correlation (*r* = 0.37, *P* < 0.05), suggesting that the effect of inversion was similar for natural objects and their silhouettes. The effect of inversion was stronger for oblique views compared with profile views for natural objects but not their silhouettes (average dissimilarity for natural objects: 0.15 for profile views, 0.18 for oblique views, *P* < 0.05; for silhouettes: 0.13 for profile and 0.15 for oblique, *P* = 0.15; Wilcoxon signed-rank test across 24 objects). We could not discern any systematic object attribute that led to these effects, save for the fact that objects that are asymmetric about the horizontal axis seem to show larger effects (Fig. 6*C*).

In summary, we conclude that the effect of silhouetting and inversion on neural responses are relatively independent of each other, but depend on object structure.

## DISCUSSION

Here, we investigated the effect of silhouetting and inversion on view invariance in monkey IT neurons across many objects tested at two views. Our main findings are *1*) view invariance is reduced by silhouetting but not by inversion; *2*) neurons with high view invariance for natural objects also show strong view invariance for silhouettes and inverted objects and generalize across silhouetting and inversion, suggesting that view invariance depends on features common to upright, silhouette, and inverted objects; *3*) neural responses to natural objects resembled that of their silhouettes early in the response and inverted objects later in the response, indicative of coarse-to-fine processing; and *4*) the effect of silhouetting and inversion depended on object structure and were independent of each other. Below, we review these findings in relation to the existing literature.

An important aspect of our study is that we have tested objects at only two views. While this certainly limits the generality of our findings, it is likely that they will generalize to other viewpoints for the following reasons. First, they are likely to hold for smaller viewpoint changes since the image itself does not change much, which could only produce greater invariance than that observed here. Second, they are likely to hold for larger viewpoint changes as well, since neural responses will then become subject to mirror confusion and again yield invariance (Rollenhagen and Olson 2000). Finally, we have found that the effect of silhouetting and inversion on shape selectivity is relatively small and independent of their effect on view invariance. This makes it likely that these effects hold across multiple views at least as long as selectivity is unaltered.

Our goal was to understand view invariance in IT neurons through systematic image manipulations. Our main finding is that silhouetting reduces view invariance but does not abolish it, suggesting that view invariance arises, at least in part, from external contours that are still present in silhouettes. It is also possible that external contours suffice to infer the three-dimensional shape, particularly when objects are more planar. This is similar to what we have reported previously (Ratan Murty and Arun 2015), but here, we have gone further to show that cells with strong view invariance on original object images also do so for their silhouettes and inverted versions (Fig. 2) and also generalize across these manipulations (Fig. 4). These observations indicate that view invariance is strongly driven by external contours and by inversion-independent features. Our findings are in agreement with the fact that humans can recognize most objects even from their silhouettes (Panis et al. 2008; Quinn et al. 2001) and when inverted (Guyonneau et al. 2006; Sekuler et al. 2004). However, our findings are by no means conclusive about the precise features, but rather provide constraints on any model of view-invariant recognition.

We have found that neural responses to natural objects resemble their silhouette version early in the response and match their inverted versions only later in the response. This stands in contrast to the expected pattern from image pixels or model V1 units, which is that upright objects resemble inverted objects more than their silhouettes. Rather, the early match between natural and silhouettes in IT neurons indicate an initial wave of processing that computes a coarse match between their external contours, making objects and their silhouettes more similar than inverted objects. The late match between natural and inverted objects indicates a subsequent wave of inversion-independent feature processing, making upright and inverted objects more similar. This is consistent with a coarse-to-fine processing sequence in IT neurons since external contours convey coarse spatial information (Sripati and Olson 2009; Sugase et al. 1999).

## GRANTS

This research was funded by a CSIR Senior Research Fellowship (to N. A. Ratan Murty), an Intermediate Fellowship from the Wellcome Trust-DBT India Alliance and the DBT-IISc partnership program (to S. P. Arun).

## DISCLOSURES

No conflicts of interest, financial or otherwise, are declared by the authors.

## AUTHOR CONTRIBUTIONS

N.A.R.M. and S.P.A. conceived and designed research; N.A.R.M. performed experiments; N.A.R.M. and S.P.A. analyzed data; N.A.R.M. and S.P.A. interpreted results of experiments; N.A.R.M. prepared figures; N.A.R.M. and S.P.A. drafted manuscript; N.A.R.M. and S.P.A. edited and revised manuscript; N.A.R.M. and S.P.A. approved final version of manuscript.
